# Virus Proteins Prevent Cell Suicide Long Enough to Establish Latent Infection

**DOI:** 10.1371/journal.pbio.0030430

**Published:** 2005-11-15

Little more than a small genome encased in protein, a virus can't reproduce without help from the cell it may ultimately destroy. After attaching to the cell membrane, a virus slips inside the cell, then co-opts its transcription and replication machinery to reproduce. Some viruses, such as the Epstein-Barr virus (EBV), enter a latent stage before they start to reproduce. During latency, the virus expresses its own genes, which help maintain the viral genome until replication begins.

In many viral life cycles, including EBV, reproduction continues until the cell bursts and releases a new crop of viruses. As a defense, cells undergo programmed cell death, or apoptosis, which protects other cells by containing the invader. But just as the host depends on apoptosis to survive, viral survival depends on preventing apoptosis. To do this, many viruses use relatives of a protein called Bcl-2 (the viral version is called vBcl-2). Though many vBcl-2–like proteins, or homologs, can trigger apoptosis, all the viral Bcl-2 homologs identified so far inhibit apoptosis during viral reproduction.

In a new study, Markus Altmann and Wolfgang Hammerschmidt use EBV as a model system to explore whether viral proteins play a role in latent infection. EBV, a tumor-causing herpes virus, targets B lymphocytes, the immune system's antibody-producing cells. While most herpes viruses encode one Bcl-2 gene, EBV encodes two, *BALF1* and *BHRF1*. *BHRF1* is known to prevent apoptosis in cells, but there is no consensus on *BALF1* function.

Altmann and Hammerschmidt created several viral mutants to probe *BHRF1* and *BAFL1* function. Interestingly, they found that blocking the function of both genes had no effect during viral reproduction. But what about the latent stage of the EBV life cycle? To investigate this question, the authors compared *BHRF1* and *BAFL1* activity with that of EBV genes expressed during latency. While 11 latency genes are expressed throughout the latent phase of EBV's life cycle, Altmann and Hammerschmidt found that both *BHRF1* and *BAFL1* act transiently—their transcripts were detected just 24 hours after infection, but not three weeks later, when the other genes were still active. [Fig pbio-0030430-g001]

**Figure pbio-0030430-g001:**
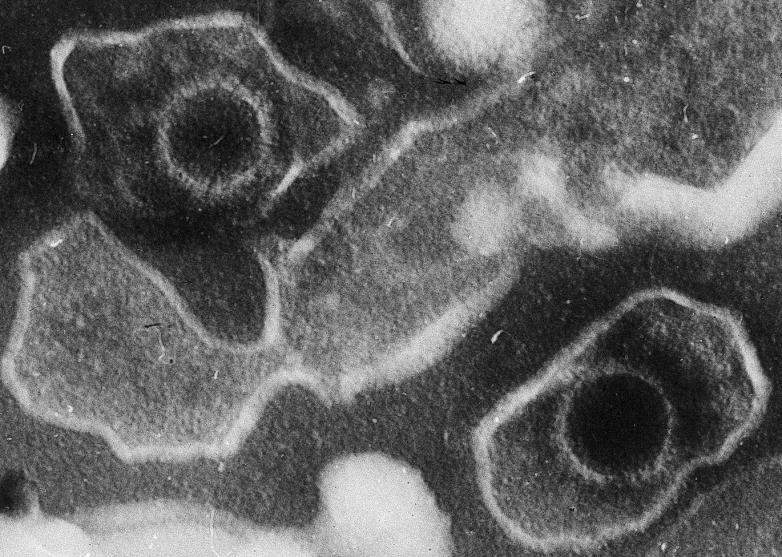
This electron microscopic image of two Epstein Barr Virus virions (viral particles) shows round capsids—protein-encased genetic material—loosely surrounded by the membrane envelope

This unexpected temporal regulation of gene expression suggested that *BHRF1* and *BAFL1* activity might initially regulate apoptosis in the infected cells. One of the genes linked to latent EBV infection, called *EBNA2*, regulates other latent genes and is required for the establishment of latency. To tease apart the effects of *EBNA2* from those of *BHRF1* and *BAFL1*, the authors infected nondividing primary B lymphocytes with mutant viruses lacking *EBNA2* in one set of experiments, and with double mutants lacking both *BHRF1* and *BAFL1* in another set. Three days after infection, some 70% of cells infected with the double-mutant virus were dying. “In stark contrast,” the authors noted, all the B lymphocytes infected with the *EBNA2* mutants—which expressed *BHRF1* and *BAFL1* right after infection—were still alive.

Once the virus is persistent in B lymphocytes, it can transform the cells into a cancerous state called lymphoblastoid cell line. This transformation did not occur when both *BHRF1* and *BAFL1* were nonfunctional. But when the authors reconstructed the double-mutant strain with just one functional gene, the virus regained its ability to transform the cells. Thus, the two genes are redundant—if one is disabled, the other can take its place.

Altogether, these results show that *BHRF1* and *BAFL1* play a critical role in establishing latent infection by preventing apoptosis until the latency genes can be activated. After the virus penetrates into the cell, either *BHRF1* or *BAFL1* keeps the cell alive initially, then *EBNA2* activates the latency genes, allowing the virus to persist and promote B lymphocyte transformation. Because vBcl2 homologs had not been directly linked to latent infection before, this study shines new light on the resources viruses use to bypass the host's defenses. Future studies can begin to explore the mechanisms regulating *BHRF1* and *BAFL1* expression, which may ultimately suggest ways to disrupt their activity and interfere with the virus's success. —*Liza Gross*

